# Long-Term Effects on Hypothalamic Neuropeptides after Developmental Exposure to Chlorpyrifos in Mice

**DOI:** 10.1289/ehp.11696

**Published:** 2008-08-22

**Authors:** Sabrina Tait, Laura Ricceri, Aldina Venerosi, Francesca Maranghi, Alberto Mantovani, Gemma Calamandrei

**Affiliations:** 1 Section of Food and Veterinary Toxicology, Department of Food Safety and Veterinary Public Health and; 2 Section of Neurotoxicology and Neuroendocrinology, Department of Cell Biology and Neurosciences, Istituto Superiore di Sanità, Rome, Italy

**Keywords:** arginine vasopressin, developmental neurotoxicity, endocrine disruptors, organophosphorus insecticides, oxytocin, prolactin

## Abstract

**Background:**

Increasing evidence from animal and human studies indicates that chlorpyrifos (CPF), similar to other organophosphorus insecticides still widely used, is a developmental neurotoxicant. Developmental exposure to CPF in rodents induces sex-dimorphic behavioral changes at adulthood, including social and agonistic responses, which suggests that CPF may interfere with maturation of neuroendocrine mechanisms.

**Objectives:**

We assessed the hypothesis that CPF affects the levels of neurohypophyseal hormones acting as modulators of social behavior in mammals, such as oxytocin (OT), arginine vasopressin (AVP), and prolactin (PRL).

**Methods:**

Pregnant female mice were orally administered with either vehicle (peanut oil) or 3 or 6 mg/kg CPF on gestational day (GD) 15 to GD18, and offspring were treated subcutaneously with either vehicle or 1 or 3 mg/kg CPF on postnatal days (PNDs) 11 to PND14. Dose levels were chosen to avoid systemic toxicity and inhibition of brain acetylcholinesterase. Offspring were sacrificed at 5 months of age, and expression of OT, AVP, and PRL was analyzed in the hypothalamus by Western blot or enzyme-linked immunosorbent assay (ELISA) analysis.

**Results:**

Both male and female mice showed dose-related enhancement of OT expression, with males presenting the more intense effect. AVP expression was significantly reduced in male mice at the higher prenatal and postnatal dose. We observed no significant effect on PRL expression in either sex. Overall, outcomes were mainly attributable to fetal exposure, whereas postnatal doses appeared to potentiate the prenatal effects.

**Conclusions:**

Our data indicate that developmental exposure to CPF may permanently interfere with specific key signaling proteins of the hypothalamic peptidergic system, with time-, dose-, and sex-related effects still evident at adulthood.

Long-term exposure to food and environmental contaminants at dose levels devoid of systemic toxicity may nonetheless affect the function of target organs. This is of particular concern for *in utero* exposure, which can affect the fetal programming of critical tissues, thus representing a key risk factor for normal growth and development.

Organophosphorus (OP) insecticides such as chlorpyrifos (CPF), conventionally considered mainly as acetylcholinesterase (AChE) inhibitors, are potential endocrine-disrupting chemicals (EDCs) (Hodgson and Rose 2007; Meeker et al. 2006). CPF is still commonly used on agricultural crops; it was also widely used for urban pest control until the United States restricted residential use because of its developmental neurotoxicity (U.S. Environmental Protection Agency 2002). The main metabolite, CPF oxon, prevents acetylcholine degradation, thus acting as cholinesterase inhibitor (Dam et al. 1999a; Mileson et al. 1998; Slotkin 2004), whereas CPF itself has a more general neurotoxic action, interfering with neural cell replication and differentiation and with synapse development and function (Casida and Quistad 2004; Crumpton et al. 2000; Dam et al. 1999a; Roy et al. 1998; Slotkin 2004).

The association between developmental neurotoxicity and OP exposure in humans is a major environmental health issue. A recent study has shown for the first time that *in utero* CPF exposure, at subtoxic doses, is related to persistent behavioral effects in children, affecting different neuropsychologic domains, such as attention and social responses (Rauh et al. 2006). Findings in rodents indicate that CPF alters the functional maturation of neurotransmitter systems such as the dopaminergic and serotonergic systems (Aldridge et al. 2004, 2005b; Dam et al. 1999b; Raines et al. 2001; Slotkin et al. 2002), thus causing persistent neurobehavioral abnormalities even at exposures well below the threshold for AChE inhibition (Barone et al. 2000; Carr et al. 2001; Dam et al. 2000; Icenogle et al. 2004; Levin et al. 2001; Qiao et al. 2002, 2003; Ricceri et al. 2003, 2006). In particular, the rat serotonergic system appears to be sensitive to developmental disruption by CPF during a selective window of exposure, from late gestation through the immediate postnatal period (Aldridge et al. 2005b). Interestingly, in mice, similarly to rats, the behavioral changes brought about by developmental CPF exposure are sexually dimorphic and involve associative functions, as well as sex-specific social responses (Ricceri et al. 2003, 2006). The long-term changes to the serotonergic system in multiple brain areas, in terms of up-regulation of serotonin [5-hydroxytryptamine (5-HT)] receptors and transporters, are in line with the behavioral alteration so far evidenced, because 5-HT is markedly implicated in modulation of social behavior patterns in both sexes (Aldridge et al. 2004; Bell and Hobson 1994; Olazabal et al. 2004; Raines et al. 2001).

These same behavioral patterns are also regulated by hypothalamic peptides acting as hormones and neurotransmitters. Oxytocin (OT) and arginine vasopressin (AVP) are abundant cyclic neuropeptides of nine amino acids, differing only in two residues, predominantly expressed in magnocellular neurons in the hypothalamic paraventricular nuclei (PVN) and supraoptic nuclei (SON); both neuropeptides are primarily involved in regulation of physiologic functions, such as smooth muscle contraction, labor and lactation onset (OT), and homeostasis of blood pressure and water resorption (AVP) (Bielsky and Young 2004; Gimpl and Fahrenholz 2001). In addition, increasing evidence points to their roles in neurobehavioral responses: OT plays a key role in regulating social recognition and parental care in mammals (Bosch et al. 2006; Gimpl and Fahrenholz 2001; Keverne and Curley 2004), whereas AVP regulates social recognition, aggression, and learning and memory (Bielsky and Young 2004; Bosch et al. 2005; Caldwell et al. 2008; Keverne and Curley 2004).

Pituitary prolactin (PRL) exerts a number of functions on mammary glands, as well as on accessory glands of the reproductive system, immune system, and so forth (Bole-Feysot et al. 1998; Cooke et al. 2004); however, hypothalamic PRL synthesis is independent from the anterior pituitary and is involved in the modulation of parental care, libido, and adaptive responses to stress (Bole-Feysot et al. 1998; Cooke et al. 2004).

In our previous work on the behavioral effects of prenatal and/or postnatal CPF exposure in mice (Ricceri et al. 2006), we evaluated two different treatment windows, the late gestational phase [gestational day (GD) 15 to GD18] and the late neo natal stage [postnatal day (PND) 11 to PND14], characterized by different central nervous system (CNS) maturational events and representing critical phases of susceptibility to CPF action in rodents (Garcia et al. 2003). That study was designed to compare the effects of prenatal and/or post-natal exposure to CPF doses within the range of fetal and childhood exposure (Gurunathan et al. 1998; Ostrea et al. 2002) on sexually dimorphic behaviors in adult mice. We found that CPF enhanced socio agonistic behaviors in males and increased responsiveness to pups in virgin females (Ricceri et al. 2006). In addition, CPF tended to reduce anxiety levels, with females more affected than males. In the present study, on the basis of the behavioral evidence collected, we investigated whether the same CPF treatment schedule, in addition to inducing signifi cant behavioral changes in exposed offspring, also had long-lasting effects on hypothalamic expression of OT, AVP, and PRL. To this end, male and female adult mice previously undergoing prenatal and/or postnatal exposure to CPF (Ricceri et al. 2006) were sacrificed at 5 months of age, and levels of OT, AVP, and PRL were evaluated in the hypothalamus by Western blot or enzyme-linked immunosorbent assay (ELISA) analysis.

We show here that developmental exposure to CPF affects the constitutive protein levels of OT and AVP, with a more evident effect in males, whereas PRL is unaffected. These data suggest that CPF interferes with maturation of neuroendocrine regulation of sex-dimorphic behavioral patterns in rodents, and support the hypothesis that CPF may act as an EDC by altering the physiologic modulatory activity of neurohypophyseal hormones.

## Materials and Methods

### Animals and treatments

All experiments on animals were performed with regard for alleviation of suffering, in accordance with European Council Directive 86/609/EEC (European Economic Community 1986) and the Italian legislation on animal experimentation. The experimental schedule has been described in detail previously (Ricceri et al. 2006). We used 30 pregnant CD1 mice: 10 vehicle controls, 10 treated with 3 mg/kg CPF (CPF3), and 10 treated with 6 mg/kg CPF (CPF6). Briefly, prenatal CPF (Chem Service, Inc., West Chester, PA, USA), dissolved in peanut oil vehicle, was administered to pregnant females by intra oral gavage [volume, 0.1 mL/kg body weight (bw)] from GD15 to GD18 (CPF3 or CPF6). Control animals received similar vehicle administration on the same schedule. Within each of the 30 litters (culled at birth to the standard size of eight pups), we treated six pups by subcutaneous injection in the nape of the neck from PND11 to PND14: one male and one female were randomly assigned to receive vehicle, one male and one female to receive CPF 1 mg/kg (CPF1), and one male and one female to receive CPF3 treatment (split-litter design). This established nine treatment groups: M0P0, M0P1, M0P3, M3P0, M3P1, M3P3, M6P0, M6P1, M6P3, where M indicates pregnant female treatment, P represents postnatal treatment to the newborn, and numbers correspond to the CPF dose in milligrams per kilogram body weight per day administered prenatally (M dose) and/or postnatally (P dose). As a result, all the pups in a litter received the same prenatal treatment but belonged to six different combinations of postnatal treatment by sex. Whole litters were weaned on PND23, maintained in same-sex pairs, and left undisturbed until the start of the behavioral studies (2 months). At 5 months of age, nine females and nine males from each of the nine treatment groups were sacrificed by decapitation, and hypothalami were excised and frozen for protein analysis.

### Sample preparation

Mouse hypothalami were lysed in 100 μL lysis buffer (20 mM Tris-HCl, pH 7.4; 150 mM NaCl; 5 mM EDTA; 1% nonyl phenoxy polyethoxy ethanol, supplemented with 1 mM phenyl-methylsulfonylfluoride) and homogenized with a 2-mL sterile syringe. After a 1-hr incubation on ice, lysates were centrifuged at 13,000 rpm, 4°C, for 20 min; two or three supernatants per treatment (depending on the size) were pooled together to obtain sufficient protein content. We determined protein concentration with the Bio-Rad Protein Assay (Bio-Rad, Hercules, CA, USA) using bovine serum albumin as standard (Sigma-Aldrich, St. Louis, MO, USA).

### Western blot analysis

We separated 50 μg total extract proteins per lane into 10% Bis-Tris NuPAGE Novex precast gels (Invitrogen, Carlsbad, CA, USA) using the prestained Novex Sharp protein standards as molecular markers (Invitrogen). The gel was then electroblotted onto a PVDF polyvinyl difluoride membrane (Bio-Rad) by semidry Trans-Blot (SciePlus, Southam, Warwickshire, UK). The blotted membranes were then washed with phosphate-buffered saline (PBS) and blocked overnight with a 5% nonfat dry milk solution (Bio-Rad). After washing with PBS containing 0.1% Tween 20 (PBST), we incubated the membranes 1.5 hr at room temperature (RT) with a polyclonal anti-PRL (Santa Cruz Biotechnology, Santa Cruz, CA, USA) or anti-AVP antibody (Chemicon, Temecula, CA, USA; < 1% cross-reactivity with OT), in PBS containing 5% nonfat dry milk. After washing with PBST, we incubated the membranes 1 hr at RT with the appropriate horseradish peroxidase (HRP)–conjugated secondary antibody (Santa Cruz Biotechnology) diluted in PBS containing 5% nonfat dry milk. The blots were visualized using a Western Blotting Luminol Reagent (Santa Cruz Biotechnology) and a VersaDoc Imaging System (Bio-Rad). We performed band densitometry with the Quantity One software, version 4.6.3 (Bio-Rad).

### ELISA analysis

For each treated pooled lysate (described above), a solution of 50 μg/mL total extract proteins in coating buffer (0.1 M NaCO_3_, 0.1 M NaHCO_3_, pH 9.5) was used to coat a 96-well flat-bottomed plate (100 μL/well) for 1 hr at 37°C and then overnight at 4°C. After a PBS wash, the wells were blocked 2 hr at RT with a 5% nonfat dry milk solution (Bio-Rad). Plates were then washed with PBST and incubated for 1.5 hr at RT with a monoclonal anti-OT antibody (Chemicon) diluted in PBS containing 5% nonfat dry milk (100 μL/well). After washing with PBST, we incubated plates 1 hr at RT with HRP-conjugated secondary antibody (Santa Cruz Biotechnology) diluted in PBS containing 5% nonfat dry milk (100 μL/well). Detection was performed with the TMB (tetramethylbenzidine) Peroxidase Substrate Kit (Vector Laboratories, Burlingame, CA, USA), reading absorbance at 450 nm with a Victor 3 Multilabel Reader (PerkinElmer, Shelton, CT, USA).

### Statistical analysis

Data are presented as mean ± SE obtained from three assay determinations for each treatment group for each sex. We conducted a preliminary global analysis of variance (ANOVA) including measurements of the three peptides using transformed data (percentages based on OT, AVP, and PRL levels of the M0P0 group for each sex), with OT, AVP, and PRL levels considered as repeated measures in the same pool of hypothalami. We then performed separate ANOVAs for each neuropeptide incorporating all contributing variables: prenatal (0, 3, and 6) and postnatal (0, 1, and 3) treatments, as well as sex. We used Tukey’s honestly significant difference test for post hoc comparisons.

## Results

CPF, at the dose levels used in this study, did not elicit systemic toxicity or weight loss in pregnant females, nor did we find any significant changes in fetal viability, weight of pups at birth, general growth, or maternal care (data not shown). We observed no signs of cholinergic intoxication at 1 and 6 hr after injection on PND11–PND14, and brain AChE activity of exposed pups was unaffected by either prenatal or postnatal CPF treatment (data not shown; for brain AChE activity values, see Ricceri et al. 2006).

The preliminary global analysis revealed a main effect of neuropeptide [*F*(2,36) = 89.85, *p* < 0.0001] and significant prenatal CPF × neuropeptide and postnatal CPF × neuropeptide interactions [*F*(4,36) = 23.33, *p* < 0.0001; and *F*(4,36) = 2.66, *p* = 0.0482, respectively], thus confirming that OT, AVP, and PRL were differentially affected by CPF treatment and that separate analyses must be performed.

### OT

**Figure 1:**
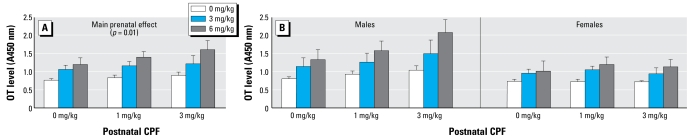
Effect of prenatal and postnatal CPF exposure on OT expression in mice. (*A*) Main effect of CPF exposure on OT peptide expression, with a significant difference (*p* < 0.01) between prenatal vehicle and 6 mg/kg bw CPF. (*B*) CPF effect on OT expression in male and female mice, with a significant difference (*p* < 0.01) in males between 6 mg/kg bw and vehicle-treated groups. Data are mean ± SE absorbance units read at 450 nm.

Overall, CPF exposure resulted in significant dose-related increase of OT expression (Figure 1A). The global ANOVA revealed a significant main effect of the prenatal treatment [*F*(2,36) = 8.48, *p* < 0.01]. Post hoc comparisons showed that OT levels were significantly higher in the CPF6-treated group than in the vehicle-treated group (*p* < 0.01; Figure 1A). As for postnatal CPF treatment, we found no significant effect and only a trend toward increased OT levels in the M6P3 group (Figure 1A). We also found a significant main effect of sex [*F*(1,36) = 12.72, *p* < 0.01]. Thus, to obtain information on different impacts of CPF in the two sexes, we performed separate analyses in males and females. The main effect of prenatal CPF was significant only in males [*F*(2,18) = 6.25, *p* < 0.01; Figure 1B]. Post hoc comparisons showed a significant difference between the higher prenatal dose, CPF6, and the vehicle-treated groups (*p*< 0.01).

### AVP

**Figure 2:**
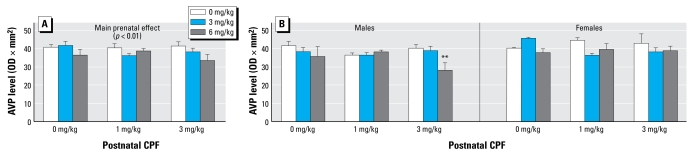
Effect of prenatal and postnatal CPF exposure on AVP expression in mice. (*A*) Main effect of CPF exposure on AVP peptide expression (*p* = 0.01). (*B* ) CPF effect on AVP expression in male and female mice. Data are mean ± SE optical density × mm^2^ as determined by electrophoresis gel densitometry. **p< 0.01 compared with M6P3 and vehicle groups.

Overall, we found a dose-dependent decrease in AVP expression in the hypothalamus after CPF treatment (Figure 2A). The global ANOVA yielded a main effect of prenatal CPF treatment [*F*(2,27) = 7.25, *p* < 0.01], whereas the postnatal treatment effect was not significant. We also found a significant effect of sex [*F*(1,27) = 6.25, *p* < 0.01]. AVP expression was markedly reduced in male mice exposed prenatally to CPF6. Post hoc comparisons performed on the interaction sex × prenatal × postnatal treatment [F(4,36) = 3.49, *p* < 0.01] showed that the hypothalamic levels of AVP were significantly decreased in M6P3 males (Figure 2B; *p* < 0.05). The effect of either prenatal or postnatal CPF was not significant in females, although a general tendency toward a decrease in AVP expression with increasing doses was apparent.

### PRL

Analysis of PRL protein expression did not reveal any significant effect after CPF exposure (either prenatal or postnatal) in both sexes. We noted an overall higher expression in females, but without any influence of the treatment (Table 1).

## Discussion

The present findings indicate that developmental exposure to subtoxic CPF doses within the range of human fetal or neonatal exposures has long-term effects on the hypothalamic protein levels of OT and AVP of mice at adulthood. Specifically, we found evidence for a long-lasting, dose-related increase in hypothalamic OT expression after prenatal and postnatal CPF exposure. AVP expression was affected to a lower extent, showing a consistent decrease only at the higher pre- and postnatal dose, M6P3, and selectively in the male sex. By contrast, no change in PRL expression was evident after CPF exposure at any dose level. Hence, developmental exposure to CPF induced a dose-, timing-, and sex-related increase of OT and decrease of AVP, without affecting PRL. Such outcomes are largely attributable to fetal (GD15–GD18) exposure to CPF at the highest dose of 6 mg/kg bw, although post-natal exposure (PND11–PND14) appears to potentiate the prenatal effects. To the best of our knowledge, this is the first study showing that CPF given during early phases of CNS development affects the constitutive levels of hypothalamic neuropeptides with a double role of neurohypophyseal hormones and neurotransmitters.

Our results indicate that both the timing of exposure and the dose level are critical for the developmental toxicity of CPF. Overall, the fetal phase emerges as the most critical window, with late neonatal phase contributing only at the highest exposure. The mouse hypothalamus becomes delineated by GD11, SON is defined from GD13, and PVN appears a day later, whereas the secretory activity of the hypothalamus-neurohypophysis system begins on GD18 (Karim and Sloper 1980). In our experiments, CPF gestational exposure spanned GD15–GD18, thus including the critical developmental window for SON and PVN neuronal precursors; as a consequence, the capacity for neuro peptide synthesis could be persistently affected. Notably, in rats, late gestational exposure to CPF (GD17–GD20) resulted in more disruption to brain functionality than did exposure during neurulation (GD9–GD12), with males more affected than females (Aldridge et al. 2005b).

Our results also indicate that postnatal exposure to 3 mg/kg bw CPF further enhanced the effect of the prenatal exposure and was more evident in male mice. Both oxytocinergic and vasopressinergic systems undergo significant postnatal changes in rodent brain; OT- and AVP-containing neurons increase with age in a similar rate for both sexes but differ in the timing of maturation. In fact, on PND1, there are more AVP- than OT-immunoreactive neurons; the main increase in AVP is between PND1 and PND8, whereas OT rises mostly during PND8–PND21 (Yamamoto et al. 2004). We carried out postnatal treatments on PND11–PND14, corresponding to a key development stage for the oxytocinergic system and a steady developmental stage for the vasopressinergic system; in fact, the oxytocinergic system was particularly affected after CPF exposure, whereas the vasopressinergic system was modulated only by the highest CPF dose. We must also consider that OT and AVP, differing by only two amino acids, may display some receptor cross-reactivity (Barberis and Tribollet 1996), thus potentially interacting during critical developmental phases. In fact, in a recent study carried out in prairie voles, manipulation of OT levels on PND1 was associated with long-lasting regional patterns of change in the AVP (V1a) receptor system, but only in males (Bales et al. 2007). Therefore, if CPF is able to interfere with the OT synthesis in PVN and SON hypothalamic neurons during critical developmental windows, it is also possible that such disruption, in turn, could have repercussions on the AVP system as well, with males more vulnerable than females. Finally, we cannot exclude that the effects on neuro hypophyseal hormones are mediated by changes in serotonergic neuro-transmission induced by CPF, previously described in rodents exposed to CPF as in the present experimental schedule (Aldridge et al. 2003; Slotkin and Seidler 2005).

We also assessed hypothalamic PRL and found it unaltered by CPF exposure. PRL belongs to a different hormone family than does OT and AVP, which derive from the same gene (Gimpl and Fahrenholz 2001). Moreover, PRL regulation follows more complex pathways not yet fully described (Cooke et al. 2004). In addition, our results in mice are consistent with previous observations showing higher hypothalamic PRL levels in females than in male rats because of the regulatory action of estradiol on brain PRL (Ben-Jonathan et al. 1996). We cannot exclude that prenatal and/or postnatal exposure to CPF elicited some reversible effect on PRL expression in mouse hypothalamus that was restored through feedback mechanisms at the time of our observation. However, our results suggest an alteration of specific neuropeptide pathways, rather than the general effect of a chemical, such as an OP insecticide, interfering with neuronal signaling.

These results lead us to reconsider the sex-dimorphic behavioral alterations observed after CPF exposure in the same set of mice (Ricceri et al. 2006). A point-to-point correlation between behavioral changes and OT and AVP levels is unfeasible, because in the present study we analyzed protein expression on tissue pools from individuals of the same experimental group. Nonetheless, the possible implication of OT and AVP in the sex-dimorphic behavioral outcomes previously observed deserves some attention. In particular, we found remarkable hyperactivity in the open-field test in male mice after pre-natal CPF exposure, either with or without an associated postnatal treatment. Moreover, CPF enhanced agonistic responses toward an unfamiliar male both in juvenile and in adult male mice. All effects in males were more marked in the highest-exposure, M6P3 group (Ricceri et al. 2003, 2006). AVP has a key role in regulating aggressive behavior in male mice: a recent study, using a mouse model of early life stress, found an inverse correlation between hypothalamic AVP levels and aggressive behavior in adult males (Veenema et al. 2007). Moreover, several pieces of evidence indicate the 5-HT system as the key regulator of the aggressive behavior in males (Bell and Hobson 1994; Simon et al. 1998); the impairment caused by CPF exposure in such regulatory system, particularly marked in males (Aldridge et al. 2004), is in agreement with our behavioral observations. In this respect, it is worth noting that a complex interaction among 5-HT, AVP, and OT has been documented in the hypothalamus of selected mouse and rat strains, as well as in transgenic mouse models, thus indicating an important role of such a signaling network in the modulation of social responses, including aggressive and affiliative behaviors (Vacher et al. 2002; Veenema and Neumann 2007; Wersinger et al. 2007).

Virgin female mice were more responsive to pups after postnatal CPF exposure (Ricceri et al. 2006). The responsiveness of nulliparous females toward pups is correlated to noradrenergic inputs in olfactory areas, independent from normal hormone regulation (Thomas and Palmiter 1997). Most mammals respond on an olfactory basis in taking care of pups; because the development of the olfactory memory in rodents is critically dependent on OT and AVP systems (Bielsky and Young 2004), the enhanced expression of hypothalamic OT could be a plausible factor in the abnormal CPF-induced onset of maternal behavior. In fact, it has been shown in rats that OT dosing triggers/elicits maternal behavior (Gimpl and Fahrenholz 2001; Kendrick et al. 1997). Finally, CPF-exposed rodents of both sexes showed reduced anxiety levels, spending more time in the open arms during plus-maze performance (Aldridge et al. 2005a; Ricceri et al. 2006). Such effect could be also related to OT increase, because this neuropeptide exerts a stress-reducing action in female mice (Gimpl and Fahrenholz 2001), where both hypothalamic and amygdaloidal OT receptors modulate anxiety levels (Bale et al. 2001).

It is currently difficult to fully assess the potential impact, if any, of the CPF-induced altered OT and AVP expression in hypothalamus, because these two neuropeptides are involved in many central behaviors, including sexual and parental ones, mediated by multiple CNS pathways. Further studies are necessary to evaluate potential alterations of oxytocinergic and vasopressinergic systems in other brain areas (amygdala, olfactory areas), including modulation in receptors expression and function, and their correlation with behavior.

In conclusion, our results demonstrate a specific, long-term impact of CPF on two neuropeptides having a critical role in behavioral processes, and confirm that late- gestational/early-prenatal CPF exposure leads to permanent gender-related effects also in mice (Aldridge et al. 2004; Dam et al. 2000; Garcia et al. 2003; Levin et al. 2001, 2002; Qiao et al. 2003, 2004). Furthermore, these results suggest a possible neuroendocrine mechanism underlying some of the sex- selective behavioral effects reported in the literature after perinatal CPF exposure, which points to a greater vulnerability of the male sex, more evident in later developmental stages (Aldridge et al. 2005a; Dam et al. 2000; Levin et al. 2001). Sex-dependent differences can indeed be a major issue in the hazard characterization of chemicals interfering with neuroendocrine pathways (Calamandrei et al. 2006). It is worth noting that OT and AVP have been implicated in autism and autism spectrum disorders, with a sex-related pattern (Carter 2007). Thus, social as well as anxiety-related behaviors might be additional sensitive targets of risk factors altering OT and AVP.

In addition, our data lend further support to CPF being considered an EDC, albeit with specific mechanisms and targets. Other OP insecticides could exert similar effects and should be further investigated at the hypothalamic–pituitary–adrenal axis level. Therefore, neuroendocrine effects, especially in susceptible developmental windows, deserve more attention in the risk assessment of OP insecticides.

## Figures and Tables

**Table 1:** Effect of CPF exposure on PRL expression in male and female mice.

Treatment	Males	Females
M0P0	30.43 ± 0.32	29.73 ± 5.84
M0P1	28.01 ± 2.63	34.49 ± 3.07
M0P3	31.10 ± 1.34	30.51 ± 3.39
M3P0	29.16 ± 1.00	37.93 ± 6.83
M3P1	27.39 ± 0.82	35.17 ± 2.16
M3P3	33.47 ± 2.58	35.91 ± 0.49
M6P0	34.99 ± 3.76	31.62 ± 1.90
M6P1	33.14 ± 4.45	29.77 ± 3.84
M6P3	28.84 ± 2.41	32.64 ± 2.43

Values are optical density × mm^2^ levels ± SE as determined by electrophoresis gel densitometry. ANOVA did not reveal any significant difference among CPF treatment groups.
